# Supplementary figures and images for: Incidence, risk factors, and prognosis of acute kidney injury in hospitalized patients with acute cholangitis
Source: PLoS One. 2022 Apr 14;17(4):e0267023. doi: 10.1371/journal.pone.0267023 (PMC9009613; doi:10.1371/journal.pone.0267023)

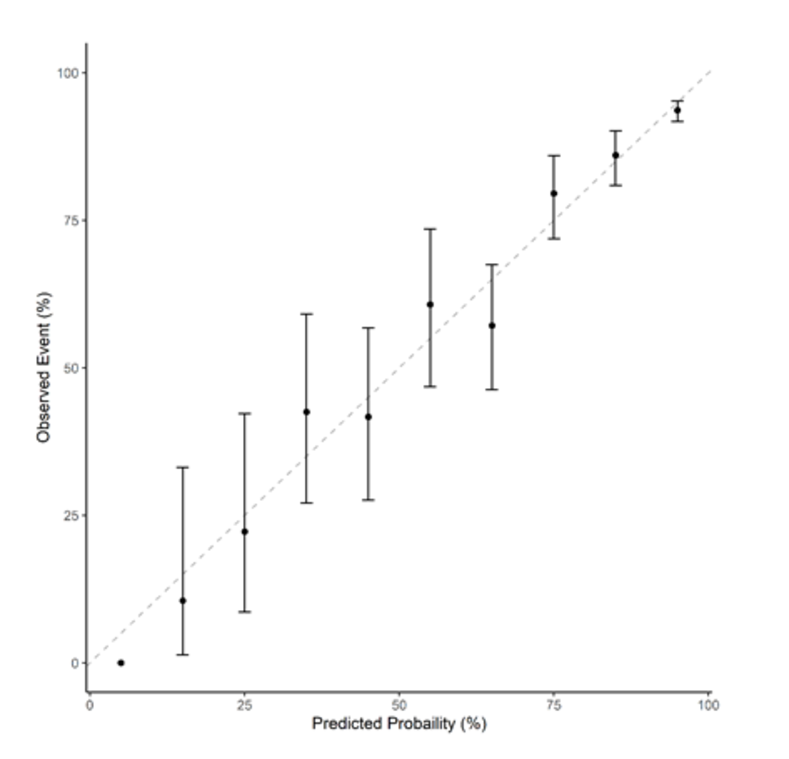

Supplement: S1 Fig — (TIF) [file pone.0267023.s001.tif]
