# Supplementary material for: Incidence, risk factors, and prognosis of acute kidney injury in hospitalized patients with acute cholangitis
Source: PLoS One. 2022 Apr 14;17(4):e0267023. doi: 10.1371/journal.pone.0267023 (PMC9009613; doi:10.1371/journal.pone.0267023)
Supplement: Attachment — Submitted filename: Responses to reviewers.docx [file pone.0267023.s004.docx]

We appreciate your positive comments. Paragraphs revised in manuscript were highlighted yellow. Belows are our response to reviewer’s comments.

5. Review Comments to the Author

Reviewer #1: Dear Editor, thank you for the privilege of reviewing this manuscript. This is a well-designed and nicely written study that describes the statistics of AKI in acute cholangitis patients. I suggest this manuscript to published in its present form.

- We appreciate your considerate comment

Reviewer #2: This is an interesting paper for several points: the use of KDIGO criteria for AKI, number of patients and the long follow up after discharge. Authors are just kindly requested to move AKI definition to the introduction

- We appreciate your critical comment. We added AKI definition according to KDIGO criteria in the section of “Introduction”. Please check this in the section of “Introduction”, line 19~22, page 3.

Reviewer #3:

**Major concerns**
1.1 Variable selection for AKI prediction model. The authors mention using forward and backward stepwise procedures to select the predictors included in the prediction model.

- We are sorry for the wrong sentence. In our research, we used backward elimination method. Incorrect sentences were revised as follow. Variables that were significant in univariate logistic analysis were included in multivariate analysis. Please check this in the section of “Statistical analysis”, line 9~10, page 5.

1.2 Can the authors comment on the reproducibility of these variable selection procedures?

- We appreciate critical comments. We totally agree your opinion for reproducibility of our variables selection procedures. We would like to open our raw data for reproducibility of variable selection procedures in open repository site. Please confirm our raw data next DOI number. The datasets of this study are available in the google drive with follow web address https://docs.google.com/spreadsheets/d/1AeASPOvS7D634L_y7E0MZrIqlEftrHus/edit?usp=sharing&ouid=112149527212455920139&rtpof=true&sd=true. We added above DOI into “Data availability statement”.

1.3 Would a different set of variables not be selected if this study was repeated in another sample? Perhaps deciding a priori to select variables known to be predictive of AKI based on prior data and fitting the model once may be produce a more reproducible model?

- We agree on your opinion. The selection of set of variables in multivariate statistical analysis might be depending on the individuality of the researchers. Also, the selection of same set of variables in other samples might result in other analysis. In addition, it is possible to select and analyze well-known variables in existing studies or variables considered important even if they are not significant without selecting a variable by a statistical method such as the backward method. However, since the purpose of this study was to explore incidence, risk factors, and prognosis of AKI in AC patients, we used known variables according to statistical criteria. Factors such as SIRS and qSOFA have been well-known as the risk of AKI occurrence although the association TG grade with AKI occurrence was unique in our study.

1.4 Also, would be more advantageous to model age in a nonlinear fashion using restricted cubic splines?
- As in your opinion, if there is a non-linear relationship between the independent variable and the dependent variable, the restricted cubic spline method is more appropriate. Therefore, in order to confirm the nonlinear relationship between age-AKI in multivariate logistic regression analysis, we additionally analyzed the risk of AKI expression risk by applying the age variable as a restricted cubic spline method. As a result, the Age-AKI relationship was judged to be a linear relationship rather than a non-linear one (refer to the figure below). Therefore, it is considered that there is no problem in maintaining the existing analysis. We would appreciate it if you would consider this.
-

2. Validation of prediction model. Did the authors use any methods to validate their prediction model such as cross-validation or bootstrapping? In the absence of any validation, can the authors comment on how this model would perform in another dataset?

- According to your opinion, the final model of AKI risk in this study was evaluated by repeated k-fold cross validation method (10 fold, 10 repeated, 75% train dataset, 25% test dataset). As a result, the model accuracy in train and test is 0.846, which is considered a relatively valid model. Above statements was described in the section of “Results”, line 12~15, page 9. This validation method was additionally described in statistical analysis. Please check this in the section of “Statistical analysis”, see 11~13, page 5.

3. Model calibration. The authors present AUROC values for discriminant analysis but it would be important to know if model calibration was also assessed.

- We agree that it is necessary to assess the predictive probabilities of AKI development models, and therefore we performed a calibration analysis. As a result, through the calibration plot, we can evaluate that the predicted probability of the model for the occurrence of AKI in AC patients was not significantly different from the actual observed probability. Please check it, line 16~17, page 9. These results (calibration plot) were presented as a supplementary file, and “Model calibration method” was added to statistical analysis. Please check this in the section of statistical analysis”, see 15~18, page 5.

Above figure was calibration plot for AKI occurrence model plotted between predicted probability and observed probability. A dotted line is a reference line with a slope of “1”. Each dot represents predicted probability and observed events by dividing the probability into 10 equal parts. Each point is distributed on a straight line with a lope of “1” suggesting consistency between observed events and predicted probability.

4. Lag time for AKI diagnosis and Time to death. Can the authors describe if there was any lag time between study entry (on date of admission) and the date of AKI diagnosis? If so, please present the median and interquartile range. Also, if any such lag time exists for some patients, how was the time pre-AKI diagnosis modelled in the Cox proportional hazards models for all-cause mortality. Was that time attributed to unexposed person-time?
- Thank you for your query. Our patient had no lag time between study entry and AKI diagnosis because we defined AKI with first laboratory finding at admission. We also described this in the section of “Materials and Methods”. Please check it line 2~4, page 4.

**Minor concerns**
1. Figure 1. In the methods, patients were admitted between January 2011 and December 2017. The Figure says 2016. Please correct.

- We are sorry for mistake. We corrected 2016 into 2017 in Figure 1

2. Figure 2. Please include the AUROC (95% CI) on the graph.

- We added AUROC (95% CI) on the graph. Please check revised Figure 2.

3. Figure 3. Please clarify what the time axis represents. Is it time from cohort entry to end of follow-up?

- We clarified what the time axis. Please check revised Figure 3.

4. Table 2. It may be better to present the multivariable odds ratios for all the variables in the table.

- We presented the multivariable odds ratios for all the variables in the table. Please check revised Table 2.

5. Table 3. In order to make inferences about the frequency of severe AKI in the different Ac severity categories, it would be better to prevent the row percentages.

- Thank you for your considerate comments. We present the row percentages in Table 3.

6. Discussion (page 10, paragraph 2). The percentages for AKI stages (56%, 28% and 16%) cannot be interpreted as AKI rates. Please revise. Also please ensure that all data presented in the discussion is also presented in the results.

- We are sorry for our mistake. We revised paragraph of “The percentage for AKI stages into “The proportion for AKI stages”. We also described this paragraph in the section of “Results”. Please confirm it, line 11, page 10.

7. Please clarify in the manuscript text what short-term and long-term mortality means.

Sorry for ambiguous description. Shor-term mortality was defined as in-hospital mortality after admission and long-term mortality 1-year mortality. Please check it, line 27, page 4.
